# Balance Evaluation Systems Test: deutsche Übersetzung, kulturelle Anpassung und erste Ergebnisse zu Gütekriterien

**DOI:** 10.1007/s00391-022-02023-9

**Published:** 2022-02-04

**Authors:** Maren Haselwander, Yannick Henes, Matthias Weisbrod, Gudrun Diermayr

**Affiliations:** 1 Fakultät für Therapiewissenschaften, SRH Hochschule Heidelberg, Maria-Probst-Str. 3, 69123 Heidelberg, Deutschland; 2 Abteilung für Allgemeine Psychiatrie, Zentrum für Psychosoziale Medizin, Universitätsklinikum Heidelberg, Voßstraße 2, 69115 Heidelberg, Deutschland; 3 Abteilung für Psychiatrie und Psychotherapie, SRH Klinikum Karlsbad – Langensteinbach, Guttmannstraße 1, 76307 Karlsbad, Deutschland

**Keywords:** Balance Evaluation Systems Test, Interkulturelle Übersetzung, Messinstrument, Validität, Reliabilität, Sturzgefahr, Balance Evaluation Systems Test, Cross-cultural translation, Outcome measures, Validity, Reliability, Fall risk

## Abstract

**Hintergrund:**

Der Balance Evaluation Systems Test (BESTest) evaluiert die Balancefähigkeit und identifiziert die dem Balancedefizit zugrunde liegenden Systeme.

**Ziel:**

Primäre Ziele waren die Übersetzung und kulturelle Anpassung des BESTest ins Deutsche. Sekundäres Ziel war die Testung der Gütekriterien.

**Methode:**

Der Übersetzungs- und Anpassungsprozess erfolgte in 7 Schritten in Anlehnung an internationale Richtlinien. Zur Testung der Gütekriterien wurden Personen mit subjektivem oder objektivem Balancedefizit eingeschlossen. Die Testung beinhaltete die Bestimmung der internen Konsistenz mittels Cronbachs α und der Kriteriumsvalidität mittels Korrelation mit der Berg Balance Scale (BBS). Die Konstruktvalidität wurde mit der Activities-Specific Balance Confidence-Scale (ABC‑D Scale) sowie anhand der Methode der bekannten Gruppen (gestürzte vs. nichtgestürzte Teilnehmende) untersucht.

**Ergebnisse:**

Von 27 Personen liegen Ergebnisse zur Testung der Gütekriterien vor. Cronbachs α beträgt 0,95 für die Gesamtskala. Der Zusammenhang der BESTest-Werte mit denen der BBS lag mit Spearmans rho bei ρ = 0,84 (*p* < 0,001) und mit denen der ABC‑D Scale bei ρ = 0,61 (*p* < 0,001). Gestürzte und Nichtgestürzte zeigten nur im Trend unterschiedliche BESTest Werte (*p* = 0,057).

**Diskussion:**

Mit dieser Arbeit steht eine von der Entwicklerin autorisierte deutsche Übersetzung des BESTest zur Verfügung. Diese vorläufigen Ergebnisse bestätigen die Reliabilität und die Validität der Originalversion.

## Abstract (en)

**Background:**

The Balance Evaluation Systems Test (BESTest) evaluates balance ability and identifies affected systems associated with balance deficits.

**Objective:**

The primary objective was the translation and cultural adaptation of the BESTest into German. Secondary objective was the preliminary evaluation of psychometric properties of the BESTest.

**Method:**

In accordance with international guidelines, the BESTest was translated and adapted by a 7-step process. To test the psychometric properties, persons with subjective or objective balance deficits were included. Internal consistency was evaluated by means of Cronbach’s alpha. The criterion validity was tested by measuring the correlation with the Berg Balance Scale (BBS). Construct validity was examined using the Activities-Specific Balance Confidence (ABC-D) scale and the known groups methods (fallers versus non-fallers).

**Results:**

A total of 27 subjects were included in the testing of the psychometric properties. Cronbach’s alpha was 0.95 for the total scale. The correlation of BESTest scores with those of the BBS revealed a Spearmen’s ρ of 0.84 (*p* < 0.001) and 0.61 (*p* < 0.001) with those of the ABC‑D scale. Fallers and non-fallers showed a trend towards different BESTest scores (*p* = 0.057).

**Discussion:**

This paper provides a German translation of the BESTest authorized by the developer. Our preliminary results confirm reliability and validity.

Im Gegensatz zu anderen standardisierten Assessments zur Erfassung der Balancefähigkeit trifft der Balance [14] Evaluation Systems Test (BESTest) nicht nur eine Aussage, ob ein Balancedefizit vorliegt, sondern unterstützt Therapeut*innen dabei, das dem Balancedefizit zugrunde liegende System zu identifizieren.

## Hintergrund

Balanceeinschränkungen und Sturzgefahr bei älteren Menschen und Menschen mit neurologischen Erkrankungen werden von Physiotherapeut*innen mit standardisierten Assessments erfasst [24]. Balance-Assessments wie die Berg Balance Scale (BBS) [3, 9], der Tinetti-Test oder der Timed-up-go-Test dienen v. a. der Prognose oder der Evaluation [27]. Der BESTest erlaubt ebenfalls eine Einschätzung der Sturzgefahr, wurde aber v. a. entwickelt, um Therapeut*innen ein Assessment zur physiotherapeutischen Diagnosestellung an die Hand zu geben [14].

Seine Entwicklerin Fay Horak legte dem BESTest die Systemtheorie von Bernstein zugrunde [4] und beschreibt 6 interagierende Systeme der posturalen Kontrolle: biomechanische Einschränkungen, Stabilitätsgrenzen, antizipatorische Haltungsanpassung, reaktive Haltungsanpassung, sensorische Orientierung und Stabilität im Gang [14].

Der BESTest zeigte in der Testung von älteren Menschen gute bis exzellente Reliabilität und Validität [1, 20]. Es liegen Werte zur Interpretation der Testergebnisse für die Balancetestung älterer Menschen vor (u. a. der Minimal Detectable Change (MDC) [1]).

Eine deutsche Übersetzung der Originalversion war bisher nicht vorhanden. Daher waren die Übersetzung und kulturelle Anpassung des BESTest ins Deutsche primäre Ziele dieser Studie. Das sekundäre Ziel war eine erste Testung der Gütekriterien des BESTest bei Menschen mit Balanceeinschränkungen.

## Methode

Die Genehmigung zur Übersetzung wurde bei der Entwicklerin Fay Horak eingeholt. Ein positives Ethikvotum der Ethikkommission der Universität Koblenz-Landau (Campus Landau) lag vor (Antrag 45). Der Übersetzungsprozess und die kulturelle Anpassung erfolgten in Anlehnung an Beaton [2]. Die folgenden 7 Schritte wurden dabei durchlaufen:

- *Schritt 1*: Vorwärtsübersetzung durch 2 voneinander unabhängige Personen (eine mit dem BESTest vertraute und in den USA promovierte Physiotherapeutin; eine professionelle Übersetzerin ohne medizinisch-therapeutischen Hintergrund),
- *Schritt 2*: Synthese der beiden Übersetzungen zu einer vorläufigen deutschen Version; Kennzeichnung diskrepanter Formulierungen für die Expert*innenkonferenz,
- *Schritt 3*: Rückübersetzung der vorläufigen Version durch 2 voneinander unabhängige Muttersprachler*innen (eine Physiotherapeutin, mit dem BESTest nicht vertraut; eine Person ohne medizinische Kenntnisse); Vergleich der Rückübersetzungen mit der Originalversion; Protokollierung von Diskrepanzen, Anmerkungen und Unklarheiten für die Expert*innenkonferenz,
- *Schritt 4*: Durchführung der Expert*innenkonferenz mit 7 Teilnehmenden (alle an der Übersetzung Beteiligten, ein Patient*innenvertreter, ein Psychologe mit methodischer Expertise und die Projektkoordinator*innen); Besprechung und Lösung aller Anmerkungen und Unklarheiten, Erstellen der vorläufigen Version,
- *Schritt 5*: Testung der vorläufigen Version im Feld,
- *Schritt 6*: Autorisierung der finalen Version durch die Entwicklerin des BESTest,
- *Schritt 7*: erste Überprüfung der Gütekriterien der übersetzten Version bei Menschen mit Balancedefiziten.

## Verständlichkeit

### Teilnehmende

Sieben Physiotherapeutinnen und ein Physiotherapeut aus Deutschland, Österreich und der Schweiz überprüften die Verständlichkeit. Die Therapeut*innen arbeiteten in 2 neurologischen Einrichtungen, in einer orthopädischen Praxis sowie 2 psychiatrischen Kliniken. Sieben Physiotherapeut*innen hatten über 10 Jahre Berufserfahrung, eine Physiotherapeut*in zwischen 2 und 5 Jahren; vier Teilnehmende hatten einen akademischen Abschluss.

### Instrumente

Mit einem Fragebogen wurden für jedes Item die Verständlichkeit der „Instruktionen für die Therapeut*innen“, „Instruktionen für Patient*innen“ sowie die Verständlichkeit des Bewertungsschemas geprüft. Die Antwortmöglichkeiten waren „Ja“ oder „Nein“. Falls „Nein“ angekreuzt wurde, wurde um Begründung gebeten.

### Ablauf

Fünf Therapeut*innen führten den BESTest mit Patient*innen durch und füllten im Anschluss daran den Fragebogen aus. Drei weitere Therapeut*innen überprüften die Textverständlichkeit ohne Patient*innenkontakt.

### Datenauswertung

Die offenen Antworten wurden gesammelt. Folgende Kategorien wurden vorab gebildet:

1. Orthographie,
2. semantisches Verständnis,
3. pragmatisches Verständnis.

Während der Auswertung wurde eine zusätzliche Kategorie eingeführt:

4. Originalquelle BESTest/Testkonstruktion.

## Gütekriterien

### Teilnehmende

Die Ad-hoc-Stichprobe wurde über eine Selbsthilfegruppe für Menschen mit idiopathischem Parkinson-Syndrom, eine physiotherapeutische Praxis sowie über ein ambulantes Rehazentrum rekrutiert. Einschlusskriterium war ein subjektiv empfundenes oder objektiv festgestelltes Balancedefizit. Ausgeschlossen wurden Personen mit kognitiven Einschränkungen.

### Instrumente zur Überprüfung der Validität

Den 6 interagierenden Balancesystemen des BESTest werden insgesamt 36 Items zugeordnet. Jedes Item wird auf einer 4‑stufigen Skala von 0 bis 3 Punkten bewertet, wobei eine höhere Punktzahl eine bessere Balancefähigkeit bedeutet. Die maximale Punktzahl beträgt 108 Punkte, wobei sich diese mit Punktwerten zwischen 15 und 21 Punkten auf die einzelnen Systeme verteilt und die Ergebnisse sowohl für die einzelnen Systeme als auch für den Gesamttest als Prozentwerte angegeben werden können [14]. Um die Kriteriumsvalidität des BESTest zu überprüfen, wurde ein beobachtungsbasiertes Instrument, die BBS, genutzt. Zur Überprüfung der Konstruktvalidität wurde ein Selbstauskunftsinstrument zur Einschätzung der Sturzangst, die deutsche Activities-Specific Balance Confidence Scale (ABC‑D Scale) als konstruktnahes Instrument gebraucht [26].

### Ablauf

Vor der Durchführung des BESTest bzw. der BBS wurden demografische Daten, inklusive Sturzhistorie, erhoben, und die Teilnehmenden wurden gebeten, die ABC‑D Scale auszufüllen. Die Reihenfolge der Testdurchführung war ausbalanciert, um Reihenfolgeeffekte zu vermeiden.

### Datenanalyse

Obwohl die Entwicklerin ein Ordinalskalenniveau beschreibt [14], wird in der internationalen Literatur mit einem intervallskalierten Niveau gerechnet [1, 19, 23]. Daher wurde ein Intervallskalenniveau des BESTest angenommen. Für alle Assessments wurden Mittelwert, Standardabweichung sowie der minimale und maximale Wert berechnet. Zusätzlich wird, um mögliche Deckeneffekte aufzuzeigen, der Prozentsatz der Teilnehmenden, die den maximalen Wert erzielten, berichtet. Die Normalverteilungsannahme wurde mithilfe des Shapiro-Wilk-Tests überprüft. Die interne Konsistenz des BESTest wurde mit Cronbachs α berechnet. Basierend auf internationalen Studien [5, 7] wurde ein α > 0,7 erwartet. Zur besseren Interpretation der Ergebnisse wird das α für ordinale Daten berichtet [12]. Die Interpretation der Werte des ordinalen α erfolgt synonym zu den Cronbachs-α-Werten.

Zur Bestimmung der Kriteriumsvalidität wurden die Werte des BESTest mit denen der BBS mithilfe von Spearmans ρ korreliert. Die Konstruktvalidität wurde mit der konstruktnahen ABC‑D Scale ebenfalls mittels Spearmans ρ berechnet. Es wird erwartet, dass eine hohe Korrelation vorliegt [14]. Außerdem wurde mithilfe der Methode der bekannten Gruppen ermittelt, ob der BESTest zwischen gestürzten und nichtgestürzten Personen unterscheiden kann. Dabei wurde angenommen, dass gestürzte Personen einen geringeren BESTest-Wert aufweisen als nichtgestürzte Personen [22]. Aufgrund der Voraussetzungsverletzung der Varianzhomogenität der Gruppen wurde der Mann–Whitney–U-Test verwendet.

Zur Prüfung der Differenzierungsfähigkeit des BESTest wurden exemplarisch Ergebnisse von 4 Personen mit unterschiedlichen Grunderkrankungen/Einschränkungen, die im Gesamttest bis auf 1 % das gleiche Ergebnis erzielten, dargestellt [14].

## Ergebnisse

### Verständlichkeit

Insgesamt lagen 75 Rückmeldungen zur Verständlichkeit der „Instruktionen für die Therapeut*innen“, der „Instruktionen für die Patient*innen“ und zur Verständlichkeit der Bewertung der Items vor.

Nach Absprache mit der Entwicklerin wurden folgende Änderungen vorgenommen:

1. Zusammenführung der „Instruktion für die Therapeut*in“ von S. 1 mit der Instruktion zur Interrater-Reliabilität auf S. 2,
2. Hinzufügen des Links für die Trainingsvideos auf S. 1,
3. Zusammenführung der „Instruktionen für Therapeut*innen und Patient*innen“ mit dem Bewertungsschema,
4. Angleichung des Bewertungstexts bei Item 16 und Item 17,
5. Übersetzung des Begriffs „tempur pad“ mit dem Begriff „Airex Balance Pad“ ins Deutsche,
6. Übersetzung von „4–12 feet“ mit „1–4 m“,
7. Übersetzung von „assistive device“ als „Stand- und Gangunterstützung durch einen Stock oder Gehbock“,
8. Übersetzung von „physical assistance“ als „manuelle Unterstützung durch den*die Therapeut*in oder ein Halten an der Bank“,
9. die Phrase „requires touch assist“ in die Bewertung des Item 20 inkludiert sowohl die Hilfe eines Stocks als auch manuelle Unterstützung durch den*die Therapeut*innen.

### Gütekriterien

Die demografischen Daten der 27 Teilnehmenden beschreibt Tab. 1. Fünfzehn Teilnehmende berichteten über einen Sturz in der Vergangenheit. Die Ergebnisse in allen 3 Balance-Assessments zeigt Tab. 2. Dabei erzielten die Teilnehmenden im Gesamt-BESTest einen Mittelwert von 80 Punkten (±20,81). In der BBS lag der Mittelwert bei 50 (±7,22) von maximal 56 Punkten. Der durchschnittliche Prozentwert der ABC‑D Scale lag bei 73 % (±20,77). Keine Person erreichte im BESTest die maximale Punktzahl; im Vergleich erzielten 5 % der Teilnehmenden in der BBS und 3,7 % in der ABC‑D Scale die maximale Punktzahl.

**Table 1:** 

Variable	Anzahl der Teilnehmenden (*n* = 27)
*Geschlecht* (m/w)	10/17
*Alter* MW (±); Min–Max	56,70 (±16,25); 24–80
*Grunderkrankung/Symptom (n)*	
Idiopathisches Parkinson-Syndrom	6
„Upper motor neuron syndrome“	8
Muskuloskeletal Erkrankung von UEX+WS	6
Rückenmarkschädigungen	2
Neuromuskuläre Erkrankungen	2
Subjektive Instabilität	3
*Sturz* (ja/nein)	15/12

*m* männlich, *w* weiblich, *MW* Mittelwert, *SD* Standardabweichung, *Min* minimaler Wert, *Max* maximaler Wert, *UEX* untere Extremität, *WS* Wirbelsäule

**Table 2:** 

Assessment	Mögliche Spanne	MW (SD)	Minimum	Maximum
*BESTest*				
Gesamt BESTest	0–108	79,59 (20,81)	31	104
I: Biomechanische Einschränkungen	0–15	9,88 (2,79)	5	14
II: Stabilitätslimits/Vertikalität	0–21	17,55 (2,29)	13	21
III: Bewegungsübergänge, antizipatorisch	0–18	12,96 (3,90)	5	18
IV: reaktive Haltungsanpassung	0–18	11,51 (5,97)	0	18
V: sensorische Orientierung	0–15	12,59 (3,14)	4	15
VI: Stabilität im Gehen	0–21	15,07 (5,77)	2	21
*BBS*	0–56	49,93 (7,22)	31	56
*ABC‑D Scale*	0–100 %	73,35 % (20,77)	13 %	100 %

*MW* Mittelwert, *SD* Standardabweichung, *%* Prozent, *BESTest* Balance Evaluation Systems Test, *BBS* Berg Balance Scale, *ABC‑D Scale* deutsche Activities-Specific Balance Confidence Scale, *I–V* System I bis System VI

**Figure 1:**
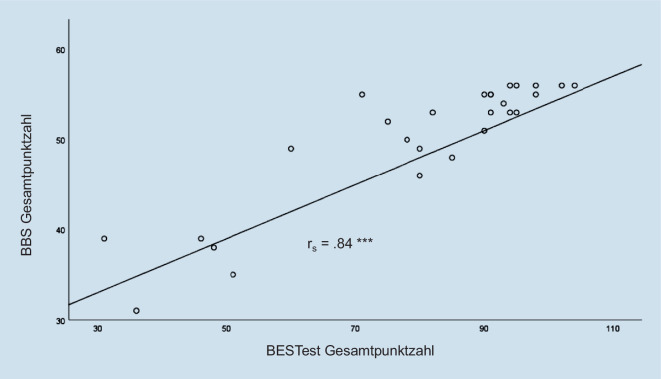

Cronbachs α zur Bestimmung der internen Konsistenz betrug 0,95 für die Gesamtskala. Das α für ordinale Daten betrug ebenfalls 0,95. Die Ergebnisse der Kriteriumsvalidität zeigt Abb. 1, wobei die Werte des BESTest mit denen der BBS hoch korrelieren (ρ = 0,84; *p* = < 0,001).

**Figure 2:**
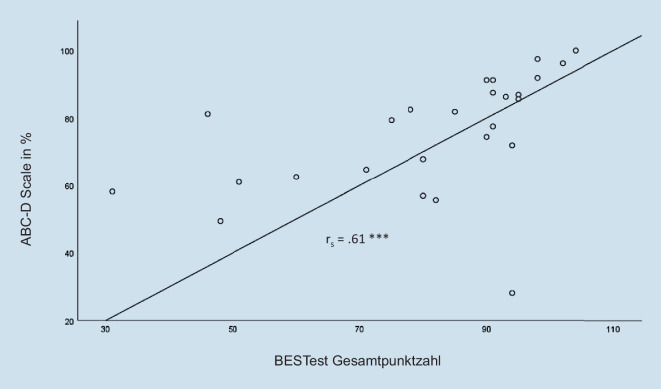

Auch die Konstruktvalidität wurde bestätigt, wobei die Werte der ABC‑D Scale mit denen des BESTest hoch korrelieren (ρ = 0,61; *p* = < 0,001; Abb. 2). Ebenso erzielten die nichtgestürzten Teilnehmenden durchschnittlich einen höheren Wert im BESTest (87,71 (±14,21) als gestürzte Personen (73,40 (±23,42); allerdings verfehlte dieser Unterschied die Signifikanzschwelle (U = 51,00, Z = −1,905333, *p* = 0,057)).

**Figure 3:**
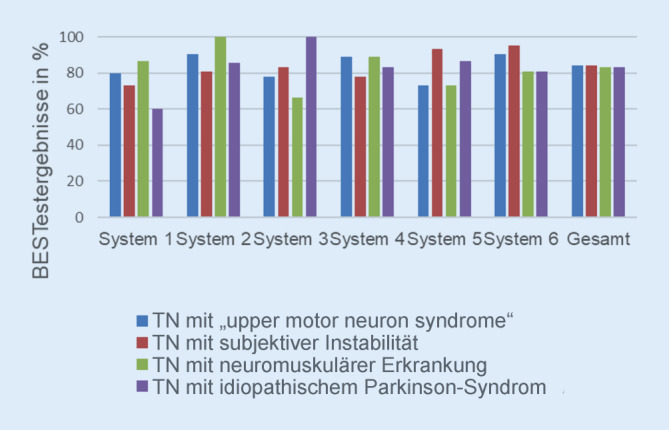

Exemplarisch zeigt Abb. 3 die Ergebnisse von 4 ausgewählten Teilnehmenden. Während sie vergleichbare Gesamtprozentwerte erreichen, weisen sie jedoch gleichzeitig unterschiedliche Ergebnisse in den einzelnen Systemen auf. Zum Beispiel erreicht die Person mit idiopathischem Parkinson-Syndrom im System 1 den niedrigsten Wert, die Person mit dem „upper motor neuron syndrome“ hingegen im System 5.

## Diskussion

Mit dieser Arbeit steht eine von der Entwicklerin autorisierte und nach internationalen Leitlinien [2] angefertigte deutsche Übersetzung des BESTest zur Verfügung. Erste Ergebnisse zu den Gütekriterien wurden anhand einer geriatrischen und neurologischen Stichprobe ermittelt.

Die Gesamtskala des deutschen BESTest zeigte für intervallskalierte und ordinale Daten exzellente Werte für die interne Konsistenz. Dies deckt sich mit der internationalen Literatur [5, 7, 25]. Die Ergebnisse aus der Bestimmung der konkurrenten Validität des BESTest mit der BBS und ABC‑D Scale bestätigen dessen Kriteriums- und Konstruktvalidität. Wie bereits in der Literatur [1, 6, 15–18, 20, 22, 25] berichtet, konnte auch für die deutsche Version – über unterschiedliche Diagnosen hinweg – ein hoher Zusammenhang mit der BBS und der ABC‑D Scale gefunden werden. Die Bestimmung der Konstruktvalidität anhand bekannter Gruppen [8] verfehlte die Signifikanz knapp (*p* = 0,057). Eine vergleichbare Studie fand ebenfalls einen Trend zur Signifikanz [22].

Der BESTest zeichnet sich darüber hinaus – im Vergleich zur BBS – durch geringere Deckeneffekte aus. Während 5 % der Stichprobe in der BBS die maximale Punktzahl erreichten, erreichte diese keine Person im BESTest. Dies deckt sich mit den Ergebnissen zur englischsprachigen Originalversion [20]. Auch Gordt fand höhere Deckeneffekte in der BBS im Vergleich zu der von ihr übersetzten Community Balance and Mobility Scale [13].

### Praktische Implikationen

Eine Übersichtsarbeit, die 66 Balance-Assessments einschloss, zeigte, dass der BESTest als einziges Assessment unterschiedliche, für die Balance verantwortliche Systeme untersucht [28]. Eine solche Untersuchung ermöglicht die Identifikation defizitärer Systeme bei vorliegenden Balanceproblemen, die in der Therapie spezifisch adressiert werden können [14]. Die exemplarische Darstellung in Abb. 3 bestätigt diese Differenzierungsmöglichkeit auch für die deutsche Version. Ein zusätzlicher Mehrwert des BESTest ist die detaillierte Testung der reaktiven Balance, die mit 5 Items standardisiert untersucht wird. In den gängigen Assessments (z. B. BBS) wird die reaktive Balance nicht oder nur sehr allgemein und ohne klare Standardisierung (z. B. Tinetti-Test) evaluiert.

Ein Nachteil des BESTest ist dessen Durchführungsdauer (ca. 30 min). In diesem Zusammenhang wurden 2 kürzere Versionen entwickelt: der Mini-BESTest [11] und der Brief-BESTest [23]. Bei älteren Menschen wird der Einsatz des Brief-BESTest empfohlen [20, 21, 29], da dieser weniger Zeit und Material in Anspruch nimmt als der Mini-BESTest und die Originalversion des BESTest [10].

### Stärken und Schwächen der Arbeit

Die Aussagekraft der Studie ist durch die geringe Stichprobengröße limitiert. Allerdings sind unsere ersten Ergebnisse mit denen aus internationalen Untersuchungen vergleichbar [5, 7, 14, 20, 25].

An der Verständlichkeitstestung nahmen 8 Therapeut*innen aus 3 deutschsprachigen Ländern sowie akademisierte und nichtakademisierte Therapeut*innen teil. Dies erleichtert die Anwendbarkeit im deutschsprachigen Berufsfeld.

## Fazit für die Praxis

- Aufgrund erster Bestätigung der Gütekriterien empfiehlt sich der Einsatz in der Praxis und Forschung.
- Der deutschsprachige BESTest ist http://www.bestest.us/files/2115/3299/3497/German_BESTest_v2.pdf online abrufbar.
